# Tobacco Smoke Exposure, Urban and Environmental Factors as Respiratory Disease Predictors in Italian Adolescents

**DOI:** 10.3390/ijerph16204048

**Published:** 2019-10-22

**Authors:** Valeria Bellisario, Pavilio Piccioni, Massimiliano Bugiani, Giulia Squillacioti, Stefano Levra, Carlo Gulotta, Giulio Mengozzi, Alberto Perboni, Elena Grignani, Roberto Bono

**Affiliations:** 1 Department of Public Health and Pediatrics, University of Turin, 10126 Turin, Italy; giulia.squillacioti@unito.it (G.S.); roberto.bono@unito.it (R.B.); 2 Pneumology and Tisiology Unit, National Health Service (ASL TO2), 10126 Turin, Italy; papiccioni@gmail.com (P.P.); maxbugiani@libero.it (M.B.); 3 Specialty School in Respiratory Diseases, University of Turin, 10126 Turin, Italy; stefanolevra@gmail.com; 4 S. Luigi Gonzaga University Hospital, 10043 Turin, Italy; gulottacarlo@gmail.com (C.G.); alberto.perboni@sanluigi.piemonte.it (A.P.); 5 Clinical Biochemistry Laboratory, A.O.U. City of Health and Science, University Hospital, 10126 Turin, Italy; gmengozzi@cittadellasalute.to.it; 6 Environmental Research Center, ICS Maugeri, Institute of Pavia, 27100 Pavia, Italy; elena.grignani@fsm.it

**Keywords:** environmental pollution, tobacco smoke exposure, childhood, spirometry, forced oscillation technique

## Abstract

Risk monitoring in childhood is useful to estimate harmful health effects at later stages of life. Thus, here we have assessed the effects of tobacco smoke exposure and environmental pollution on the respiratory health of Italian children and adolescents using spirometry and the forced oscillation technique (FOT). For this purpose, we recruited 188 students aged 6–19 years living in Chivasso, Italy, and collected from them the following data: (1) one filled out questionnaire; (2) two respiratory measurements (i.e., spirometry and FOT); and (3) two urine tests for Cotinine (Cot) and 15-F_2t_-Isoprostane (15-F_2t_-IsoP) levels. We found a V-shape distribution for both Cotinine and 15-F_2t_-IsoP values, according to age groups, as well as a direct correlation (*p* = 0.000) between Cotinine and tobacco smoke exposure. These models demonstrate that tobacco smoke exposure, traffic, and the living environment play a fundamental role in the modulation of asthma-like symptoms (*p* = 0.020) and respiratory function (*p* = 0.007). Furthermore, the results from the 11–15-year group indicate that the growth process is a protective factor against the risk of respiratory disease later in life. Lastly, the FOT findings highlight the detrimental effects of tobacco smoke exposure and urbanization and traffic on respiratory health and asthma-like symptoms, respectively. Overall, monitoring environmental and behavioral factors in childhood can provide valuable information for preventing respiratory diseases in adulthood.

## 1. Introduction

Childhood is a crucial stage of life during which harmful lifestyle habits can be easily and unconsciously acquired. This can lead to changes in metabolic pathways and unfavorable health effects in adulthood [1,2]. For example, monitoring hazard conditions and lifestyle habits during growth has been shown to be a useful parameter to estimate different risky conditions later in life, such as cardiometabolic diseases and obesity [3]. Furthermore, deleterious lifestyle habits are often worsened by exposure to environmental pollution and tobacco smoke, factors to which children and young adults are commonly exposed worldwide [4,5].

While adults avail themselves of different strategies to counteract these risk factors, children who live, play, and study in urbanized areas and/or are in close contact with smokers often become forcibly exposed. Moreover, when, for some reason, the immune system becomes partially compromised, some clinical manifestations such as lower respiratory tract infection, ear infection, and lifelong cardiovascular risk are further worsened [6]. The respiratory system is considered a primary target of air pollution. Since children breathe a proportionately greater volume of air than adults and they have smaller caliber airways, they are more likely to develop inflammation-related illnesses and increase their overall level of oxidative stress [7,8,9]. In addition, as children tend to spend more time outdoors, they are more likely to experience small airway obstruction in the presence of high levels of air pollution [10]. The respiratory health of children can also be compromised by passive and active exposure to tobacco smoke. In fact, tobacco smoke is still the main preventable risk factor for respiratory, allergic, and cardiovascular disease, as well as for cancer [11]. At earlier stages of life, passive exposure to smoke or the beginning of active addiction to tobacco can be particularly damaging. This can be ascribed to the fact that individuals are more vulnerable to the harmful effects of tobacco during early stages of growth. In this regard, mounting evidence suggests that tobacco smoke exposure in early puberty may have a negative impact on health across generations [12,13]. This is particularly important in Italy, where over 20% of 15-year-old adolescents smoke at least once a week and more than 13% smoke every day [11,14,15,16].

In this scenario, we aimed to assess behavior towards some of the most widespread and harmful determinants of lifestyle habits and respiratory health, such as passive and active tobacco smoke and environmental pollution in relation to age in a large population of children and adolescents in Italy. Finally, we sought to identify possible alterations in respiratory functions using spirometry and the Forced Oscillation Technique (FOT). This can allow the identification of differences between the two breathing measurement techniques, among a different range of ages, and between different environmental conditions, including exposure to tobacco smoke.

## 2. Materials and Methods 

### 2.1. Study Participants

For the study, 204 students were recruited from elementary, middle, and high schools of Chivasso, a small urbanized town close to Turin, Italy. Chivasso is inhabited by 26,976 people (on 1 January 2018, according to the Italian Institute of Statistics), and it extends over 51.24 km^2^, with a density of 526.48 inhabitants/km^2^. To recruit the subjects, the following criteria were adopted: a) living in or nearby Chivasso and b) being aged between 6 and 10 years (elementary schools), 11 and 15 years (middle schools), and 15 and 19 years (high schools). Because the subjects were underage, parents or guardians of children were asked to sign an informed consent for study enrollment according to the Helsinki Declaration. Sampling was carried out from January to March 2016 according to a pre-established timetable. At the end of the samplings, after the evaluation of the respiratory results, 188 subjects were selected. For each subject, we collected the following data:

1. Questionnaire: Questions selected from the most extensive SIDRIA questionnaire [16], as described previously [17], were administered to each subject enrolled. This information was used to establish individual and clinical features (i.e., age, weight, height, Body Mass Index –BMI-, gender, residence, hobbies, therapies, and health conditions). Questions on tobacco smoke and urbanization included a parental and subjective evaluation of the exposure (absent, low, moderate, or high). The questionnaire was also structured to gain in-depth knowledge of personal lifestyle habits of the subjects and to gather information on the main asthma-like symptoms, such as asthma attacks, wheeze with breathlessness, current use of treatments for asthma, current hay fever/nasal allergies, waking with chest tightness, being woken by shortness of breath, and being woken by coughing [18].
2. Spirometry measurements: These were expressed as maximal expiratory flow–volume curves to establish forced vital capacity (FVC), forced expiratory volume in the first second (FEV_1_), maximal expiratory flows at peak 50%, 25%, and among 25–75% of FVC (PEF, FEF_50_, FEF_25_, FEF_25-75_) and the FEV1/FVC ratio. The instrument (CPFS/D, MGC Diagnostics Corporation, St Paul, MN, USA) was calibrated daily with a 3 L syringe. After a brief training, the measurements were carried out in accordance with the current ATS/ERS standards [19] and repeated until the volume variability did not exceed 150 mL for at least 2 times in order to comply with both within- and between-maneuver criteria [20].
3. Respiratory mechanics: These were measured by FOT by means of a Resmon Pro FULL device (Restech, Milan, Italy). This method is noninvasive and employs small-amplitude pressure oscillations superimposed on the normal breathing, not requiring the performance of respiratory maneuvers [21]. A couple of measurements of at least 10 breaths each were obtained from each individual. The quality of breath was assessed through a specific algorithm contained inside the device and subsequent mathematical evaluation. Resistance and reactance obtained at a frequency of 5 Hz were used for the analysis.
4. Morning urine spot: This test was performed to measure the following parameters:
  - Cotinine. Cotinine measurements were carried out to objectively quantify the passive and active exposure to tobacco smoke. Cotinine levels were also regarded as a possible inductor of oxidative stress (OS) imbalance [22,23]. Urine samples were prepared for analysis as previously described [10,20,24]. Gas chromatography mass spectrometry (GC-MS) analysis was performed using an Agilent Technologies 6890 GC, interfaced to a 5973 MSD Inert Agilent mass spectrometer. The MS operated in electron impact and SIM mode. The limit of detection (LOD) and limit of quantification (LOQ) were 0.01 μg mL^−1^ and 0.02 μg mL^−1^, respectively. The coefficient of variation (CV), calculated to test repeatability, was below 5% for both Cotinine and the internal standard;
  - 15-F_2t_-Isoprostane (15-F_2t_-IsoP). 15-F_2t_-IsoP was measured to quantify OS by the ELISA technique using a specific microplate kit (Oxford, MI, USA) according to the manufacturer’s instructions. To achieve better accuracy in the competitive ELISA method, each sample was diluted 1:4. The procedure is described in more detail elsewhere [25,26]; and
  - Creatinine (Crea). Crea quantification was performed by the kinetic Jaffè procedure in order to normalize the excretion rate of Cotinine and 15-F2t-IsoP [20].
5. Statistical analyses: They were all carried out using the Stata 14 Statistical Package (Stata Corp LP, Lakeway Drive, TX, USA). In univariate analysis, the variables in ordinal or interval scale were compared between gender and age classes through the non-parametric Kolmogorov–Smirnov 2 sample equality-of-distributions test and the Kruskal–Wallis equality-of-populations rank test. The frequency differences were tested with Pearson’s chi-squared test. Differences with a *p* < 0.05 were considered significant. To analyze the determinants of 15-F_2t_-IsoP, multiple linear regression analysis was performed using Box–Cox-transformed [27] 15-F_2t_-IsoP as the dependent variable. Height, age (6–10, 10–15, or >15-year groups), log Cotinine, and smoking exposure (recorded as yes or no) were used as predictive variables. In all models, variables were retained when they reached a level of 5% significance. To assess the effect of covariates on lung function parameters (measured through spirometry and FOT), we compared the findings of the spirometric parameters with the Global Lung Function Initiative (GLI) reference values [28], assuming as cut-off of “normal” values the lower 10% confidence limit of normality (LLN), as recommended by GLI authors. A sub-sample of asymptomatic subjects not exposed to tobacco smoke was selected from the whole group to calculate the reference values for FOT still missing in a well-stabilized form. This was achieved through multiple regression analysis of Box–Cox-transformed resistance and reactance calculated at a frequency of 5 Hz as dependent variables, selecting age, height, weight, and gender (female as reference value) as independent variables. The limits of normal test variability were computed following GLI recommendations. For spirometric values, the normal values were defined by comparing them with the lower limits of normality (LLN), while FOT and oscillatory resistances were compared with elastance and the upper limits of normality (ULN). A set of multiple logistic regression analyses were performed using the abnormality of findings as dependent variable, smoking, and traffic exposure as predictors, and age, gender, and BMI as confounders. A *p* value ≤ 0.05 (two-tailed) was considered significant in all tests. All variables that were not significant at the 5% level and not influencing other parameters were excluded.

### 2.2. Compliance with Ethical Standards

All procedures performed in this study involving human participants were in accordance with the ethical standards of the local Ethics Committee of “*San Luigi Gonzaga*” Hospital (session on 11 March 2015, number 27/2015) and with the 1964 Helsinki declaration and its later amendments or comparable ethical standards.

## 3. Results

The study was circumscribed to 188 collaborative subjects who had provided valid results with respect to the respiratory parameters analyzed. Table 1 shows the main anthropometric characteristics of the subjects according to gender (top) or age group (6–10, 11–15, and >15 years) (bottom), in order to highlight whether there were differences in respiratory variables [18,29] and in OS levels [30,31,32], principally because the population was almost heterogeneous in terms of development stages but also in terms of age. 

The Kruskal–Wallis test showed no significant differences between genders for the anthropometric variables tested, whereas it detected significant differences for biologic and lung-function markers for all variables among the different age groups (Table 1). The BMI, categorized according to the IOTF criteria, indicates that over 70% of subjects had a normal BMI, while 21% were overweight or obese. A V-shape trend (Table 1, second part) was found in oxidative stress levels according to age groups (decrease in the 11–15 group, *p* = 0.00) while no significant differences were found for sex. Among the 188 students, 14 reported being active smokers (7.4%), 41 passive smokers (21.7%), and 134 non-smokers (70.9%). Table 1 also reports the means and standard deviations (SDs) of the following lung function parameters: FVC, FEV_1_, maximal expiratory flows at peak 50%, 25%, 25–75% of FVC, and inspiratory, expiratory and total resistance, as measured by FOT at 5 Hz of frequency. All these parameters proved to be homogeneous in the three age groups (KS test = NS). Alike, no significant differences in respiratory parameters were found between males and females. Finally, Table 1 shows the means and SDs of 15-F_2t_-IsoP and Cotinine, expressed as ng/mg of Crea according to gender and age group.

**Figure 1:**
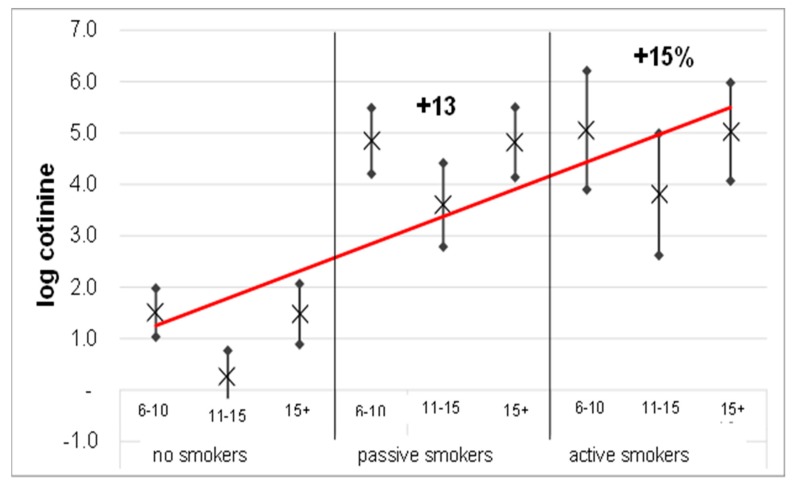
Multiple non-linear regression between log Cotinine (dependent variable) and tobacco smoke exposure, adjusted and stratified for the three age groups. For each groups the figure reported the mean (×), the predictive margins (•) and the regression between log cotinine and tobacco smoke exposure (red line).

Multiple non-linear regression performed (Table 2) on the entire population shows that the log Cotinine values, stratified and adjusted for age group, oxidative stress levels and BMI, display a V-shape trend (Figure 1). 

In particular, the analysis shows a direct and linear regression (*p* = 0.000) between Cotinine and exposure to tobacco smoke, with an increase of 13% and 15% for passive and active exposure, respectively (passive B = 1.17, CI = 95% (1.01–1.34); active B = 1.19, CI = 95% (0.97–1.41)) if compared to non-smokers.

Moreover, the V-shape trend is maintained even with the stratification for tobacco smoke exposure, with an overall 12% decrease in the >15 age group (*p* = 0.050) in comparison with the other age groups.

**Figure 2:**
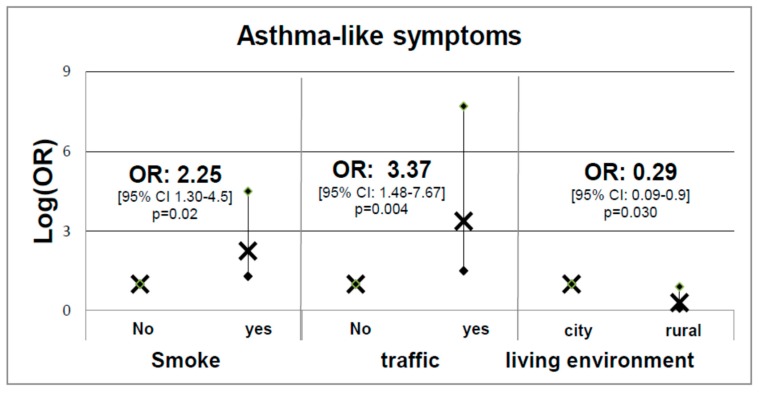
Logistic regression analysis using asthma-like symptoms as the dependent variable and tobacco smoke exposure, traffic, and living environment as independent variables, adjusted for age and gender. For each groups the figure reported the mean (×) and the predictive margins (•).

The study participants were also asked to provide answers to questions selected from the SIDRIA questionnaire [16], as described previously [17], with the aim to establish potential relationships between respiratory health, quantified through respiratory flow parameters (dependent variable), and symptoms and some risk factors such as tobacco smoke exposure, traffic, and living environment (independent variables). Using normal limits, 15 subjects had non-normal total resistance at 5 Hz values (R5-tot) (7.9%). In particular, Figure 2 illustrates the logistic regression analysis results obtained using asthma-like symptoms as the dependent variable and tobacco smoke exposure, traffic, and living environment as independent variables, adjusted for age and gender. 

**Figure 3:**
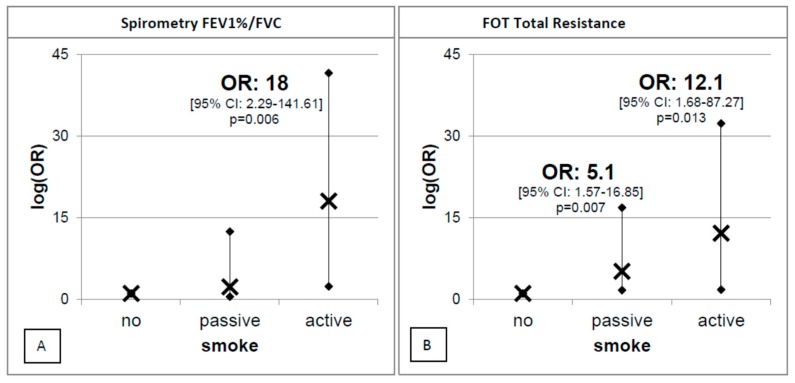
Logistic models with the FEV1/FVC ratio (**A**) and the forced oscillation technique (FOT) total resistance obtained at a frequency of 5 Hz (**B**) as the dependent variable, and tobacco smoke exposure, traffic, and living environment as independent variables, adjusted for age and gender. For each groups the figure reported the mean (×) and the predictive margins (•).

Furthermore, Figure 3 shows the logistic model with the FEV_1_/FVC ratio (on the left) and FOT R5-tot (on the right) as dependent variables, and tobacco smoke exposure, traffic, and living environment as independent variables, adjusted by age and gender. Both models clearly show that tobacco smoke exposure, traffic, and living environment play a role in the modulation of asthma-like symptoms and respiratory function.

With regard to the first model depicted in Figure 2, asthma-like symptoms were more frequently found in children exposed to tobacco smoke (OR = 2.25 (95% CI: 1.30–4.50); *p* = 0.020) or living in high-traffic areas (OR = 3.37 (95% CI: 2.29–141.61); *p* = 0.004). In contrast, living in rural areas seems to have a moderate protective role in terms of respiratory health in the subjects considered (OR = 0.29 (95% CI: 0.09–0.90); *p* = 0.030). The negative role of tobacco smoke is also confirmed in the second GLM model (Figure 3), which shows how FEV_1_/FVC and FOT parameters are negatively influenced upon tobacco smoke exposure as this latter increases substantially the FEV_1_/FVC ratio (OR = 18 (95% CI: 2.29–141.61); *p* = 0.006) but only in active smokers. Furthermore, Figure 3B shows how tobacco smoke exposure worsen significantly FOT R5-tot (OR = 5.1 (95% CI: 1.57–16.85); *p* = 0.007) and active smokers (OR = 16.8 (95% CI: 1.68–87.27); *p* = 0.013) (Figure 3B), indicating a better sensitivity of FOT with regard to early damage to respiratory pathways. No significant relationships were detected between non-normal FVC and FEV_1_ and smoke exposure, traffic, and living environment.

## 4. Discussion

In this study, we aimed to investigate some aspects of youth health through the analysis of specific determinants of health risk factors such as age, tobacco smoke exposure, and air pollution. To this end, we evaluated the respiratory and health status in a group of Italian children and adolescents using age, tobacco smoke exposure, environmental living, oxidative stress, and body composition (BMI) as independent variables. We also explored respiratory health, measured objectively by spirometry and FOT and subjectively by a questionnaire, using passive and active tobacco smoke, intensity of automotive traffic, and urbanization level as independent variables. 

Oxidative stress concentrations and BMI levels were found to be closely related to age groups, as already found in our previous works [20,33], but in the deepened analyses we did not find statistical correlations with lung parameters and the other variables. 

Figure 1 display an evident V-shape distribution of log Cotinine values sub-grouped according to the three age groups, which shows a substantial decrease in the intermediate age group. In particular, the findings relative to the intermediate age group (11–15 years) underscore the importance of the role played by the growth process in the respiratory health of a mature individual. This particular role in growth-period processes is indeed characterized by a particular hormonal change triggering the activation of enzyme systems, and this seems to give an acceleration of enzyme systems and to protective metabolism. This, in turn, enhances the hormonal and metabolic action, likely lowering the concentration of the analytes measured in this study and yet to be investigated with future longitudinal studies [34,35]. 

With regard to the effects induced by exposure to tobacco smoke we show a direct correlation between Cotinine load and tobacco smoke exposure in the three age groups (Figure 1). Furthermore, we show that tobacco smoke exposure is not only an inductor of asthma-like symptoms (Figure 2, left-hand side) but also a factor that increases both the FEV1/FVC ratio (Figure 3A) and the total resistance measured by FOT (Figure 3B). In particular, the harmful effect of smoke on respiratory functions is clearly evidenced by both the spirometry and FOT results. Overall, these two techniques appear to be extremely useful and reliable for the assessment of respiratory function in school-age subjects, showing a reduction in respiratory function in active smokers, which is particularly alarming considering the young age of the subjects involved in the study. On the other hand, the impact of secondhand smoke on functional parameters is only revealed by FOT, which appears to be a more sensitive technique than spirometry in detecting small differences between non-smokers and passive smokers. Moreover, FOT seems to be more easily tolerated by children than spirometry, and thus much easier to perform, in good agreement with a previous report [36]. FOT also appears to be more sensitive than spirometry in early detection of increased airway resistance and, more generally, of precocious damage to the respiratory system, making this technique particularly suited to screen young populations. 

## 5. Conclusions

Exposure to traffic and urban residences are important determinants of asthma-like symptoms, which can also be induced by a number of other independent risk factors. Altogether, our findings indicate that monitoring childhood growth trajectories can provide us with important information that can be used to conceive and structure early interventions aimed at preventing the development of health risk in adolescents, a strategy that has already proven successful in reducing the incidence of age-related chronic illnesses such as respiratory or cardiovascular disease and obesity [37]. 

A second and perhaps more important lesson is that a thorough analysis of tobacco smoke exposure at young ages is clearly needed to prevent the harmful effects of urban environmental pollution on respiratory health [38]. In addition, active and passive exposure to tobacco smoke, to which young people are particularly sensitive, is another important inductor of respiratory disease that needs to be taken into account, especially in view of the fact that in adolescents the onset of smoking habit is still growing [11]. 

One of the strengths of this study is the number of subjects involved and the wide age range of the study participants: 188 subjects, from 7 to 19 years old. A limitation of the study is the qualitative estimation of exposure to motor vehicle traffic and the type of urban or rural residence, which was based on subjective responses to the questionnaire, and the lack of information on allergies. 

Overall, passive and active tobacco smoke exposure in adolescents must be strongly reduced through a powerful educational action aimed at spreading awareness, in preventive terms, and with the utmost clarity on various smoke-related risks such as respiratory and cardiovascular diseases. Furthermore, we have to stress the need for policies aimed to counteract the influence of smoking parents and grandparents on adolescents [39]. 

Finally, from a clinical and functional standpoint, our study underscores that even exposure to urban environmental pollution can affect respiratory health since childhood. Thus, strategies for reducing air pollution in heavily urbanized areas should improve health outcomes while reducing heath care expenditures.

## Figures and Tables

**Table 1:** Anthropometric characteristics of the subjects according to gender (**top**) or age group (**bottom**).

**Individual Characteristics**		**Total** **(*n* = 188)**	**Male** **(*n* = 103)**	**Female** **(*n* = 85)**	***p*** **Value** **(KS/KW test)**
**Age (years)** Mean ± S.D.		12.9 ± 3.8	12.9 + 3.9	12.9 + 3.6	0.719
**Height (m)** Mean ± S.D.		1.6 ± 1.7	1.6 + 1.9	1.5 + 1.3	0.729
**Weight (Kg)** Mean ± S.D.		50.1 ± 17.3	52.7 + 19	46.8 + 13.8	0.090
**BMI** Mean ± S.D.		19.6 ± 3.8	19.9 + 4	19.1 + 3.4	0.229
**BMI** **IOTF** **No. (%)**	Underweight	17 (9%)	7 (6.8%)	10 (11.6%)	
	Normal weight	132 (69.8%)	76 (73.8%)	56 (65.1%)	
	Overweight	27 (14.3%)	10 (9.7%)	17 (19.8%)	
	Obese	12 (6.9%)	10 (9.7%)	2 (3.5%)	
**Smoking habits** **No. (%)**	No	134 (70.9%)	71 (68.9%)	63 (73.2%)	
	Passive	41 (21.7%)	20 (19.4%)	21 (24.4%)	
	Active	14 (7.4%)	9 (11.7%)	5 (5.8%)	
**Isoprostane (ng/mg Crea)** Mean ± S.D. (Min–Max)		4.5 ± 4.7 (0.2–38.8)	4 ± 3.8 (0.8–17.7)	5.1 ± 5.7 (0.2–38.8)	0.06
**Cotinine (ng/mg Crea)** Mean ± S.D. (Min–Max)		102 ± 196.9 (0.1–1730.9)	92.6 ± 151.4 (0.1–742.5)	115.5 ± 241 (0.1–1730.9)	0.15
**FVC** Mean ± S.D.		3.5 ± 1.5	3.8 ± 1.7	3.1 ± 1.2	**0.00**
**FEV1** Mean ± S.D.		3.1 ±1.3	3.4 ± 1.5	2.7 ± 0.9	**0.00**
**FEF25** Mean ± S.D.		5.4 ± 2.3	6 ± 2.7	4.8 ± 1.4	**0.00**
**FEF50** Mean ± S.D.		3.9 ± 1.7	4.3 ± 2.1	3.6 ± 1.1	**0.00**
**FEF25–75** Mean ± S.D.		3.5 ± 1.6	3.9 ±1.7	3.2 ± 1.1	**0.00**
**FEV1/FVC** Mean ± S.D.		0.8 ± 0.1	0.9 ± 0.04	0.8 ± 0.08	**0.011**
**R5 tot** Mean ± S.D.		4.2 ± 1.7	4.2 ± 1.9	4.2 ± 1.4	**0.01**
		**6–10 years old** **(*n* = 74)**	**11–15 years old** **(*n* = 53)**	**15 + years old** **(*n* = 61)**	***p*** **Value** **(KS/KW test)**
**Isoprostane (ng/mg Crea)** Mean ± S.D.		4.7 ± 5.3	3.8 ± 4.2	5.1 ± 4.5	**0.00**
**Cotinine (ng/mg Crea)** Mean ± S.D.		74.6 ± 109.7	33.2 ± 111.6	196.7 ± 284.7	**0.00**
**FVC** Mean ± S.D.		2.2 ± 0.4	3.7 ± 1.3	4.9 ± 1.3	**0.00**
**FEV1** Mean ± S.D.		1.9 ± 0.3	3.3 ± 1	4.3 ± 1.2	**0.00**
**FEF25** Mean ± S.D.		3.7 ± 0.6	5.7 ± 1.7	7.4 ± 2.4	**0.00**
**FEF50** Mean ± S.D.		2.7 ± 0.5	4.1 ± 1.2	5.4 ± 1.9	**0.00**
**FEF25–75** Mean ± S.D.		2.4 ± 0.5	3.7 ± 1.1	4.9 ± 1.8	**0.00**
**FEV1/FVC** Mean ± S.D.		0.9 ± 0.1	0.9 ± 0.1	0.9 ± 0.1	**0.00**
**R5 tot** Mean ± S.D.		5.7 ± 1.3	4 ± 1.3	2.8 ± 0.7	**0.00**
**X5 tot** Mean ± S.D.		−1.8 ± 0.8	−1.2 ± 0.7	0.9 ± 0.3	0.54

BMI = Body Mass Index; IOTF = International Obesity Task Force; FVC= Forced Vital Capacity; FEV1 = Forced expiratory Volume in the First Second; FEF= Maximal Expiratory Flows; FEV1/FVC = FEV1/FVC ratio; Crea = Creatinine; R5 tot= total resistance; X5tot = total reactance

**Table 2:** Multiple non-linear regression parameters.

Independent Variables		Predictive Margins (95% C.I.)	*p*
**Total sample**	No	1.04 (0.72–1.36)	**0.000**
	Passive	1.17 (1.01–1.34)	
	Active	1.19 (0.97–1.41)	
**No** **smokers**	6–10 years old	1.5 (1–2)	**0.050**
	11–15 years old	0.3 (−0.2–0.8)	
	15 + years old	1.5 (0.9–2.1)	
**Passive smokers**	6–10 years old	4.8 (4.2–5.5)	
	11–15 years old	3.6 (2.8–4.4)	
	15 + years old	4.8 (4.1–5.5)	
**Active** **smokers**	6–10 years old	5.1 (3.9–6.2)	
	11–15 years old	3.8 (2.6–5)	
	15 + years old	5.0 (4.1–6)	
